# The BH3 Mimetic Obatoclax Accumulates in Lysosomes and Causes Their Alkalinization

**DOI:** 10.1371/journal.pone.0150696

**Published:** 2016-03-07

**Authors:** Vasileios A. Stamelos, Natalie Fisher, Harnoor Bamrah, Carolyn Voisey, Joshua C. Price, William E. Farrell, Charles W. Redman, Alan Richardson

**Affiliations:** 1 Institute for Science and Technology in Medicine, Keele University, Stoke-on-Trent, Staffordshire, United Kingdom; 2 School of Pharmacy, Keele University, Keele, Staffordshire, United Kingdom; 3 University Hospital of North Midlands, Stoke-on-Trent, Staffordshire, United Kingdom; Innsbruck Medical University, AUSTRIA

## Abstract

Obatoclax belongs to a class of compounds known as BH3 mimetics which function as antagonists of Bcl-2 family apoptosis regulators. It has undergone extensive preclinical and clinical evaluation as a cancer therapeutic. Despite this, it is clear that obatoclax has additional pharmacological effects that contribute to its cytotoxic activity. It has been claimed that obatoclax, either alone or in combination with other molecularly targeted therapeutics, induces an autophagic form of cell death. In addition, obatoclax has been shown to inhibit lysosomal function, but the mechanism of this has not been elucidated. We have evaluated the mechanism of action of obatoclax in eight ovarian cancer cell lines. Consistent with its function as a BH3 mimetic, obatoclax induced apoptosis in three cell lines. However, in the remaining cell lines another form of cell death was evident because caspase activation and PARP cleavage were not observed. Obatoclax also failed to show synergy with carboplatin and paclitaxel, chemotherapeutic agents which we have previously shown to be synergistic with authentic Bcl-2 family antagonists. Obatoclax induced a profound accumulation of LC-3 but knockdown of Atg-5 or beclin had only minor effects on the activity of obatoclax in cell growth assays suggesting that the inhibition of lysosomal function rather than stimulation of autophagy may play a more prominent role in these cells. To evaluate how obatoclax inhibits lysosomal function, confocal microscopy studies were conducted which demonstrated that obatoclax, which contains two basic pyrrole groups, accumulates in lysosomes. Studies using pH sensitive dyes demonstrated that obatoclax induced lysosomal alkalinization. Furthermore, obatoclax was synergistic in cell growth/survival assays with bafilomycin and chloroquine, two other drugs which cause lysosomal alkalinization. These studies explain, for the first time, how obatoclax inhibits lysosomal function and suggest that lysosomal alkalinization contributes to the cytotoxic activity of obatoclax.

## Introduction

Obatoclax is a drug that belongs to a class of compounds called BH3 mimetics which promote apoptosis through activation of the intrinsic apoptosis pathway. We have studied this class of drugs in ovarian cancer cells because there is a clear need to develop new therapies for this disease. Current treatment comprises debulking surgery and chemotherapy, which often comprises the combination of carboplatin and paclitaxel. The majority of patients initially respond, but disease reoccurrence and the development of drug resistance limits long term survival and only 30% of patients survive 5 years beyond diagnosis [1]. Obatoclax antagonizes the Bcl-2 family of proteins (Bcl-2, Bcl-X_L_, Bcl-w, Mcl-1 and A1/Bfl-1 [2]), which suppress apoptosis. BH3 mimetics sensitize cells to pro-apoptotic stimuli and we have previously demonstrated that two BH3 mimetics, navitoclax and ABT-737, are synergistic with carboplatin in ovarian cancer cells [3, 4]. However, synergy between BH3 mimetics and carboplatin was only observed in a subset of the cells we evaluated. One potential explanation for this is the expression of Bcl-2 family members, such as Mcl-1 or A1/Bfl1, that do not bind ABT-737 and navitoclax with high affinity. In contrast, obatoclax binds to all the Bcl-2 family apoptosis inhibitors with comparable affinities [5] suggesting that it may be more broadly effective than ABT-737 or navitoclax. Several studies have found that exposure to obatoclax induces apoptosis in several cancer cell types as well as showing synergy with chemotherapeutic agents or molecularly targeted agents including several kinase, HDAC and proteasome inhibitors (reviewed in ref [6]). This has led to its evaluation in 19 clinical trials [7].

Although obatoclax can act as a BH3 mimetic, it clearly possesses additional pharmacological activity. Obatoclax retains significant activity in cells depleted of Bak and/or Bax, key effectors in the intrinsic apoptosis pathway [8–10]. Previous reports have claimed differing effects on cell cycle progression in various cell lines including G_1_ [11, 12] or S phase [8, 13] arrest or lack of arrest in a particular phase [13, 14]. The cytotoxic activity of obatoclax has been proposed to be due to the drug’s ability to act as an anion ionophore [15], to increase expression of the Trail receptor DR5 [16] and to inhibit mTORC1/2 [17]. However, numerous reports have claimed BH3 mimetics, particularly obatoclax, induce autophagy. These claims are mostly based on the profound accumulation of autophagosomes and LC3 induced by these drugs [10, 18–24]. A mechanistic rationale exists for this because BH3 mimetics can liberate the autophagy initiator Beclin from sequestration by Bcl-2 family members [25]. This has raised the possibility that autophagy might contribute to the cytotoxic activity of obatoclax, although it could equally represent a cell survival response to a toxic insult. In support of the former interpretation, several reports have claimed that obatoclax (in combination with kinase inhibitors and HDAC inhibitors) induces a toxic form of autophagy [26–28], or that obatoclax induces necroptosis as a result of the assembly of a necrosome signaling complex on the accumulating autophagic vesicles [14, 29]. Furthermore, most [18, 23, 24, 29], but not all [10] reports suggest that repressing the expression of key autophagy genes such as those encoding Beclin, Atg-5 and Atg-7 reduces the cytotoxic activity of obatoclax suggesting that autophagy contributes to this process. In contrast, Schwartz-Roberts and co-workers [30] showed that obatoclax inhibits lysosomal activity by reducing the level of cathepsins and this is associated with an increase in the number of lysosomes and accumulation of LC3. This argues obatoclax inhibits the end stage of autophagy. This observation is consistent with the foregoing data because blockade of lysosomal activity could cause accumulation of autophagosomes and nucleate the formation of a necrosomes and trigger necroptosis. Crucially, however, the mechanism by which obatoclax inhibits lysosomal function has not been reported. Despite these uncertainties in its predominant mechanism(s) of action, obatoclax has recently been proposed as an exemplar for a novel strategy to treat cancer by developing therapeutics that induce “autophagic cell death” [31].

We set out to evaluate the mechanism of action of obatoclax in more detail. Building on previous work showing synergy between *bona fide* BH3 mimetics [3, 4] and carboplatin or paclitaxel, we first evaluated whether obatoclax was also synergistic with these chemotherapeutic agents. Obatoclax showed fundamental differences from our previous studies with BH3 mimetics, it was not synergistic with the chemotherapeutic agents and in some cell lines was even antagonistic. In contrast obatoclax induced accumulation of LC-3 in all eight cell lines tested, consistent with the drug stimulating autophagy or inhibiting lysosomal function. However, knockdown of two key genes that regulate autophagy had minor effects on the activity of obatoclax, suggesting that inhibition of lysosomal function was more likely to contribute to the drug’s activity. Measurement of cathepsin activity in intact cells confirmed that obatoclax inhibited proteolytic activity. To address how obatoclax inhibited lysosomal activity, we measured the subcellular localization of obatoclax and found that it accumulated in lysosomes. Studies with pH sensitive dyes revealed that obatoclax caused lysosomal alkalinization. Obatoclax was also synergistic in cell growth/survival assays with other agents that promote lysosomal alkalinization, resulting in apoptosis. Thus these data suggest that a major mechanism of action of obatoclax is to cause lysosomal alkalinization and explain for the first time how obatoclax inhibits lysosomal function.

## Materials and Methods

All cell lines (obtained from ATTC, NCI and ECCAC) except from Ovcar-3 were grown in Roswell Park Memorial Institute medium (RPMI 1640; Sigma) supplemented with 10% fetal bovine serum (FBS; Sigma), 2mM L-Glutamine (Sigma) and 50U/ml penicillin/streptomycin (Sigma). Ovcar-3 cells were grown in the same medium supplemented additionally with insulin (Sigma) and 1mM sodium pyruvate (Sigma).

Cell growth/survival and drug combination studies were conducted as previously described using 18 drug concentrations [3]. Briefly, cells in 96 well plates were exposed to drug for 72 hours in 100 μl growth medium. Surviving cells estimated by staining with 0.4% sulforhodamine B (SRB). Data were analyzed using Graphpad Prism to fit a 4 parameter Hill equation. For drug combination studies, cells were exposed to18 different concentrations of obatoclax and either carboplatin, paclitaxel or chloroquine combined at a ratio of the single agent IC_50_s to obtain a complete dose-response curve Data were analyzed using Graphpad Prism to fit a 4 parameter Hill equation and CI values were calculated as described and are reported at fraction affected = 0.5 [32]. For experiments where the schedule of drug administration was altered, cells were sequentially exposed to the drugs at a ratio of their single agent IC_50_s. Cells were exposed to one drug for 48 hours, washed twice with 100 μl PBS and then exposed to the second drug for a further 48 hours before staining with SRB. C.I. values were calculated using IC_50_ and Hill coefficients derived from parallel experiments in which cells were exposed to the individual drugs for the same time period as in the combination.

To assess viability using trypan blue, 12-well plates were seeded with 50,000 cells/well and after 24 hours obatoclax was added at 5 multiples of the IC_50_ previously measured in cell growth/survival assays (Table 1). After 72 hours, the cell population was trypsinized, resuspended in 0.4% Trypan Blue in PBS and the viable and non-viable cells were counted with a hemocytometer.

**Table 1:** Potency of obatoclax against 8 ovarian cancer cell lines. Cells were treated with obatoclax for 72 h and the surviving cell number estimated by staining with SRB. Results are presented as the mean ± S.D. of n independent experiments.

Cell line	IC50 (nM)
A2780	59 ± 30 (n = 17)
cisA2780	72 ± 39 (n = 15)
Ovcar-3	35 ± 28 (n = 11)
Ovcar-4	140 ± 130 (n = 10)
Ovcar-5	78 ± 29 (n = 15)
Ovcar-8	97 ± 35 (n = 15)
Igrov-1	150 ± 78 (n = 15)
SkOv-3	220 ± 92 (n = 9)

3-D multicellular ovarian cancer spheroids were established from Ovcar-8 cells using the “hanging droplet” technique. A 20 μl drop of Ovcar-8 cell suspension (1.25 x 10^5^ cells/ml) in growth medium were placed on the lid of 48-well plates. Sterile water was added to each well to minimize evaporation. Lids were inverted over the wells and incubated for 7 days, after which the spheroids were treated with 5 μl obatoclax at 6 multiples of the IC_50_ measured in cell growth assays. After 72 hours spheroids were collected into 20 μl of PBS and ATP measured by addition of 25 μl Cell titre glo (Promega) according to the manufacturer’s instructions.

For colony formation assays, Ovcar-8 cells were seeded in 6-well plates as described above and treated with obatoclax at 6 multiples of the IC_50_ measured in cell growth assays for 48 hours. Cells collected by trypsinization, counted and a between 100 and 100,000 cells reseeded per well of a new 6 well plate. After 15 days colonies were stained with methylene blue (0.5% w/v in 50% ethanol) and colonies > 1 mm counted.

siRNA transfection was performed as previously described [33] using 20 nM siGENOME SMARTpools (Dharmacon) in the presence of 0.1% Dharmafect-1. qRT-PCR was performed using SybrGreen (Abgene) according to the manufacturer's instructions using a 7900HT Fast Real-Time PCR machine. Primers for Atg5 and Beclin-1 autophagy genes were designed using the NCBI Entrez Gene database and the Primer3 software. The sequences for primers used were as follows: forward1, Beclin, 5′-AGATGCGTTATGCCAGAC- 3′; reverse1, Beclin 5′-GATTGTGCCAAACTGTCCAC- 3′; forward2, Beclin, 5′- CAGGCTGAGGCTGAGAGAC- 3′; reverse2, Beclin 5′-TTCAGCTCATCATCCAGCTC - 3′; forward1, Atg5, 5′- GCATCAAGTTCAGCTCTTCC - 3′; reverse1, Atg5 5′- GATGGACAGTGCAGAAGGTCT- 3′; forward2, Atg5, 5′-CAGATGGACAGTTGCACACA - 3′; reverse2, Atg5 5′- TGTTGGCTGTGGGATGATAC- 3′. Amplification efficiency was determined by serial dilution of the template and the measured efficiency used to calculate %knockdown.

For western blotting, cells were collected, washed with PBS, and then lysed with a modified RIPA [34]. Protein was measured using the BCA assay (Sigma). Samples were analyzed using 3–12% BisTris gels (NuSep) according to the manufacturer's instructions and the separated proteins were transferred to nitrocellulose membranes (Hybond-ECL, GE Healthcare, Buckinghamshire, UK) or PVDF. LC3 was detected with mAb clone 2G6 (Nanotools; 2G6 detects LC3-I and LC3-II), PARP with a polyclonal rabbit antibody (Cell signaling technologies # 9542) and GAPDH with mAb clone 6C5 (Millipore).

For cell cycle analysis, 6-well plates were seeded with 1 x 10^5^ cells and the next day treated with obatoclax for 48 hours at 37°C. Cells were collected by trypsinization, fixed with 70% ethanol and stained with propidium iodide (final concentration 50μg/ml) containing RNAse. Flow Cytometry data were acquired using a BD FACsort flow cytometer and data were analyzed with Cyflogic. To measure Annexin V and PI staining, Ovcar-5 or Ovcar-8 cells were exposed to Obatoclax and/or Bafilomycin for 48 hours. Floating and adherent cells were collected by trypsinization, combined and stained with Annexin-V/FITC and propidium iodide (Miltenyi Biotech) according to the manufacturer’s instructions before analysis by flow cytometry.

To measure caspase activity, 5000 cells were seeded per well of a 96 well plate in 80 μl growth medium. The next day, 20 μl obatoclax was added at the indicated concentration. After 30 hours caspase activity was measured using Caspase-glo 3/7 (Promega) using according to the manufacturer’s instructions.

Cathepsin activity was assessed by measuring the release of 7-Amino-4-methyl coumarin (AMC) from the cathepsin substrate Z-Phe-Arg-AMC as previously described [35]. 20,000 Ovcar-5 cells/well were seeded in a 96 well plate. Cells were exposed to obatoclax (120nM) for periods up to 58 hours, washed twice with phenol red free DMEM (not supplemented with fetal calf serum) and subsequently incubated for a further 12 hours in the presence of the substrate Z-Phe-Arg-AMC (100 μM; Bachem) and either solvent, E-64d (100μM) or obatoclax (120 nm). Fluorescent AMC was measured (λ_Ex_ = 360 nm, λ_Em_ = 450 nm) and the results normalized to relative cell number by staining the cells with SRB.

For confocal microscopy studies, Ovcar-4, Ovcar-5 or Ovcar-8 cells were loaded with either 1 μM lysosensor green DND-189 (1 μM for 1 hour) or lysosensor yellow-blue DND-160 (1 μM, 7 min) (Life technologies) or lysotracker blue (75 nM, 1 hour) at 37°C. Cells were washed briefly in Liebovitz’s L15 medium (Life technologies) containing 10% FCS and then exposed to 120 nM obatoclax in L15/FCS. For video studies, images were collected from drug or control cells every minute for up to 1 hour. To measure the colocalization of obatoclax with LAMP1, 10,000 Ovcar-5 cells were treated with 6μl of Bacmam 2.0 LAMP-2 GFP or RFP reagent (Life technologies). After 16 hours, cells were exposed to 120nM obatoclax for 1 hour and co-localization assessed by confocal microscopy. Lysosensor Green and Lamp-2-GFP were excited with the 488 nm laser line, obatoclax with the 573 nm laser line and Lysotracker Blue with the 405 nm laser line.

## Results

**Fig 1:**
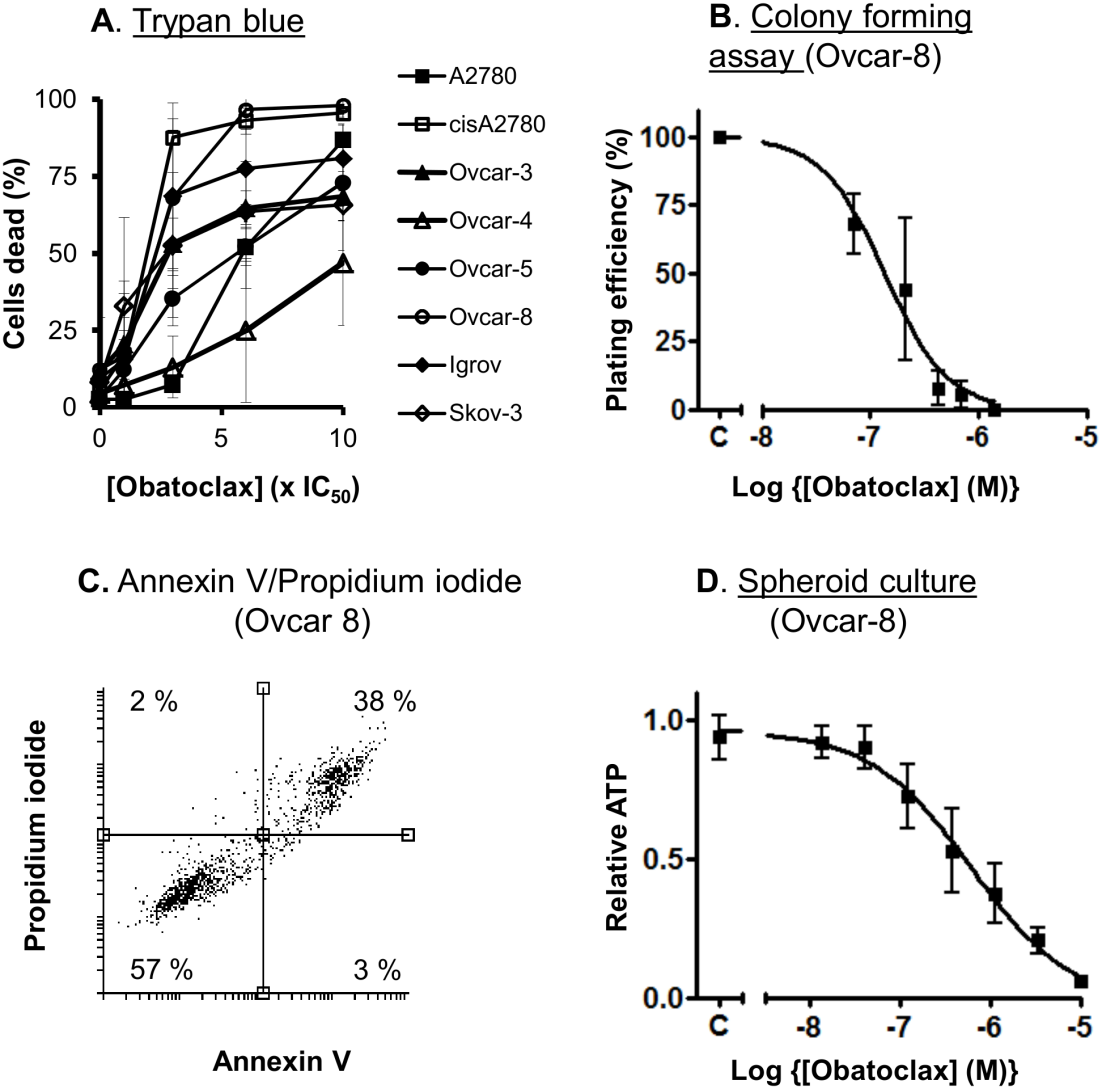
Obatoclax causes cell death. **A**. Cells were exposed to obatoclax at the indicated multiple of IC_50_ that had previously been determined in cell proliferation assays (Table 1). After 72 hours, the fraction of counted cells (mean ± S.D., n = 2–4) that were stained with trypan blue was determined. In all of the cell lines, there was a significant increase in dead cells (*P* < 0.05, paired t-test) in all of the cell lines. **B**. Ovcar-8 cells were exposed to the indicated concentration of obatoclax for 48 hours. Cells were collected by trypsinization, replated and the number of surviving cells estimated by a colony forming assay. The results (mean ± S.D., n = 3) are expressed as a fraction of the plating efficiency measured in cells treated with vehicle. **C**. Cells were exposed to 1 μM obatoclax for 48 h, collected by trypsinization, stained with Annexin V and propidium iodide and analysed by flow cytometry. The result shown is representative of 3 experiments. **D**. Ovcar-8 cells were grown as spheroids for 1 week then exposed to the indicated concentration of obatoclax for 72 hours. The spheroids were lysed and ATP measured. The results (mean ± S.D., n = 3) are expressed as a fraction of the ATP measured in cells treated with vehicle.

We first evaluated the activity of obatoclax in cell growth studies using 8 ovarian cancer cell lines. We have previously observed that authentic BH3 mimetics such as ABT-737 and navitoclax have relatively weak single agent activity in these cells [3, 4]. In contrast, obatoclax inhibited the growth of cultures of all the cell lines with nanomolar potency (Table 1) even in cells resistant to carboplatin, eg cisA2780, Ovcar-5 and Ovcar-8 cells. To distinguish between inhibition of cell proliferation or induction of cell death, the viability of cells following exposure to a range of concentrations of obatoclax was assessed by staining with trypan blue (Fig 1). Obatoclax led to a significant increase in the fraction of dead cells, although a smaller proportion of the Ovcar-3, Ovcar-4 and Skov-3 cells than the other cells were killed even at the highest concentration tested. In contrast, flow cytometry experiments did not suggest pronounced cell cycle arrest (S1 Fig) although a substantial increase in sub-G_1_ fraction was observed ins some cells, particularly A2780, cisA2780 and Ovcar-8 cells. To confirm that obatoclax was cytotoxic, we assessed its activity in colony forming assays using Ovcar-8 cells (Fig 1B). Cells were exposed to a range of concentrations of obatoclax, collected by trypsinization and replated. The exposure to obatoclax reduced the plating efficiency (IC_50_ = 140 nM), confirming that obatoclax caused cell death. At the highest concentration of drug used (1.4 μM) no surviving cells were observed. This was, at least in part, due to apoptosis, because obatoclax increased staining with Annexin V (Fig 1C). We confirmed the activity of obatoclax in a more physiological model using Ovcar-8 cells grown as spheroids (Fig 1D) and measured intracellular ATP and the nanomolar cytotoxic activity of obatoclax was retained (IC_50_ = 790 ± 90 nM, n = 5).

**Fig 2:**
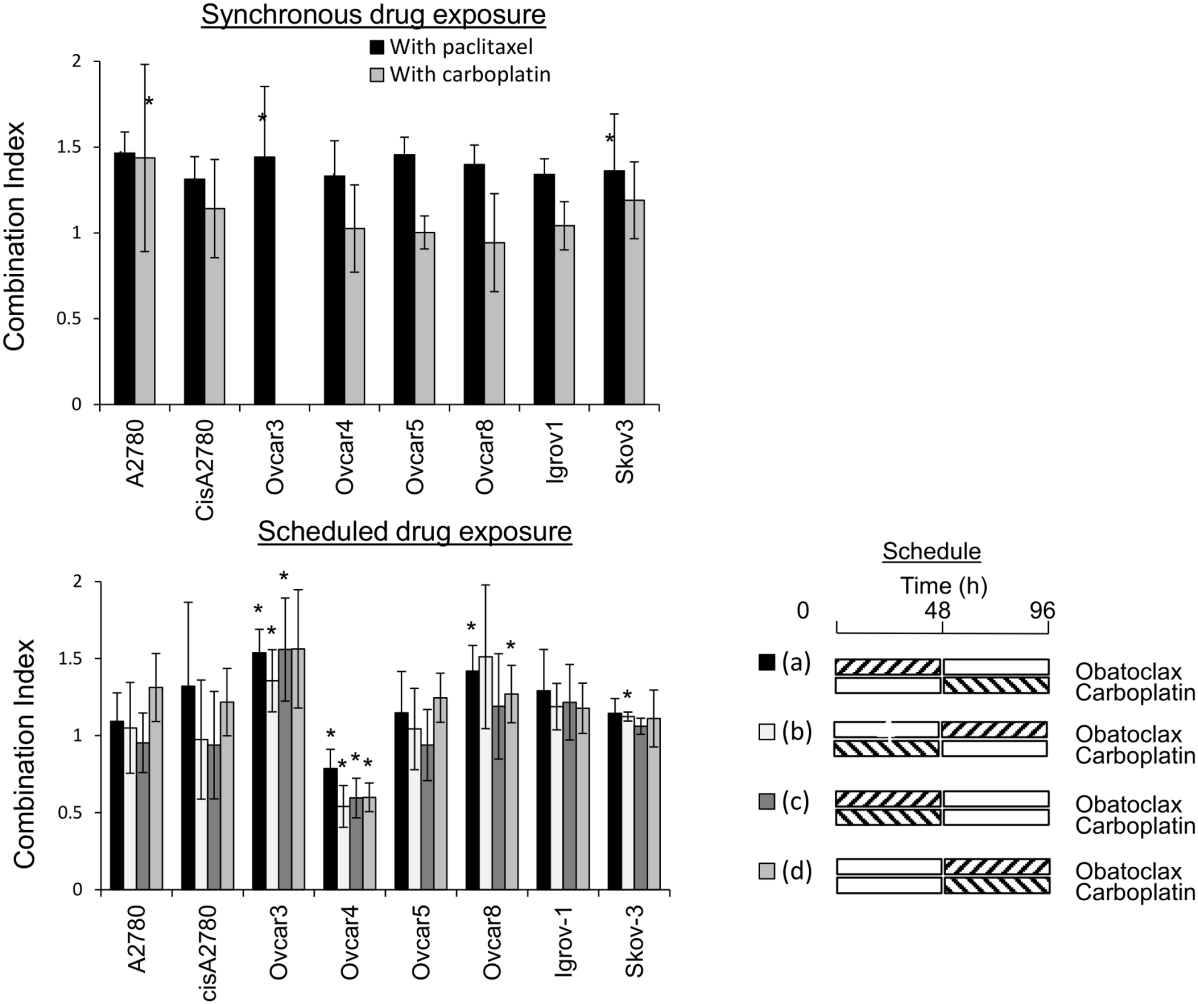
Combinations of obatoclax with carboplatin or paclitaxel. **A**. Cells were treated with obatoclax and either carboplatin or paclitaxel, combined at the ratio of the IC_50_s determined in cell growth/survival assays with the individual drugs. After 72 hours, the surviving cell number was estimated by staining with SRB and the combination index calculated as described in the methods. The results are expressed as the combination index at fraction affected = 0.5 (mean ± S.D., n = 3–10) and were significantly different from 1.0 where indicated (*, *P* < 0.05, t-test using Welch’s correction). **B**. Scheduled combinations of obatoclax and carboplatin. Cells were exposed to: a) obatoclax for 48 hours then carboplatin for 48 hours; b) carboplatin for 48 hours, then obatoclax for 48 hours; c) carboplatin and obatoclax for 48 hours then culture medium for a further 48 hours; d) culture medium for 48 hours, then carboplatin and obatoclax for 48 hours. In each case, the cells were treated with 18 different concentrations of carboplatin and obatoclax combined at the ratio of their IC_50_s determined in experiments with the individual drugs. Combination indices were determined at fraction affected = 0.5 mean ± S.D., n = 3–4) and were significantly different from 1.0 where indicated (*, *P* < 0.05, t-test using Welch’s correction).

We anticipated that if the activity of obatoclax primarily reflected inhibition of Bcl-2 family proteins, it would be synergistic with carboplatin because we have previously demonstrated that ABT-737 and navitoclax, authentic BH3 mimetics, are synergistic with both carboplatin and paclitaxel [3, 4]. However, when cells were simultaneously exposed to carboplatin and obatoclax, additivity rather than synergy was observed (Fig 2A). Notably, we found no evidence for synergy between carboplatin and obatoclax in Igrov-1 cells, cells in which we have previously observed marked synergy between carboplatin and ABT-737 [3]. We previously found additivity between paclitaxel and ABT-737 in cell growth assays [4] but in a further departure from the behavior of an authentic BH3 mimetic, when the cells were exposed simultaneously to obatoclax and paclitaxel, significant antagonism was observed in some cell lines (Fig 2A). We have previously observed that synergy between ABT-737 and carboplatin is dependent on the order of drug addition with exposure to carboplatin prior to ABT-737 being the most effective schedule of those evaluated [3]. Consequently, we investigated different schedules of drug administration in which the eight ovarian cancer cell lines were treated with obatoclax either before or after treatment with carboplatin and compared with synchronous treatment (Fig 2B). Altering the schedule of administration did not lead to significant synergy, and only modest synergy was observed in one cell line (Ovcar-4).

**Fig 3:**
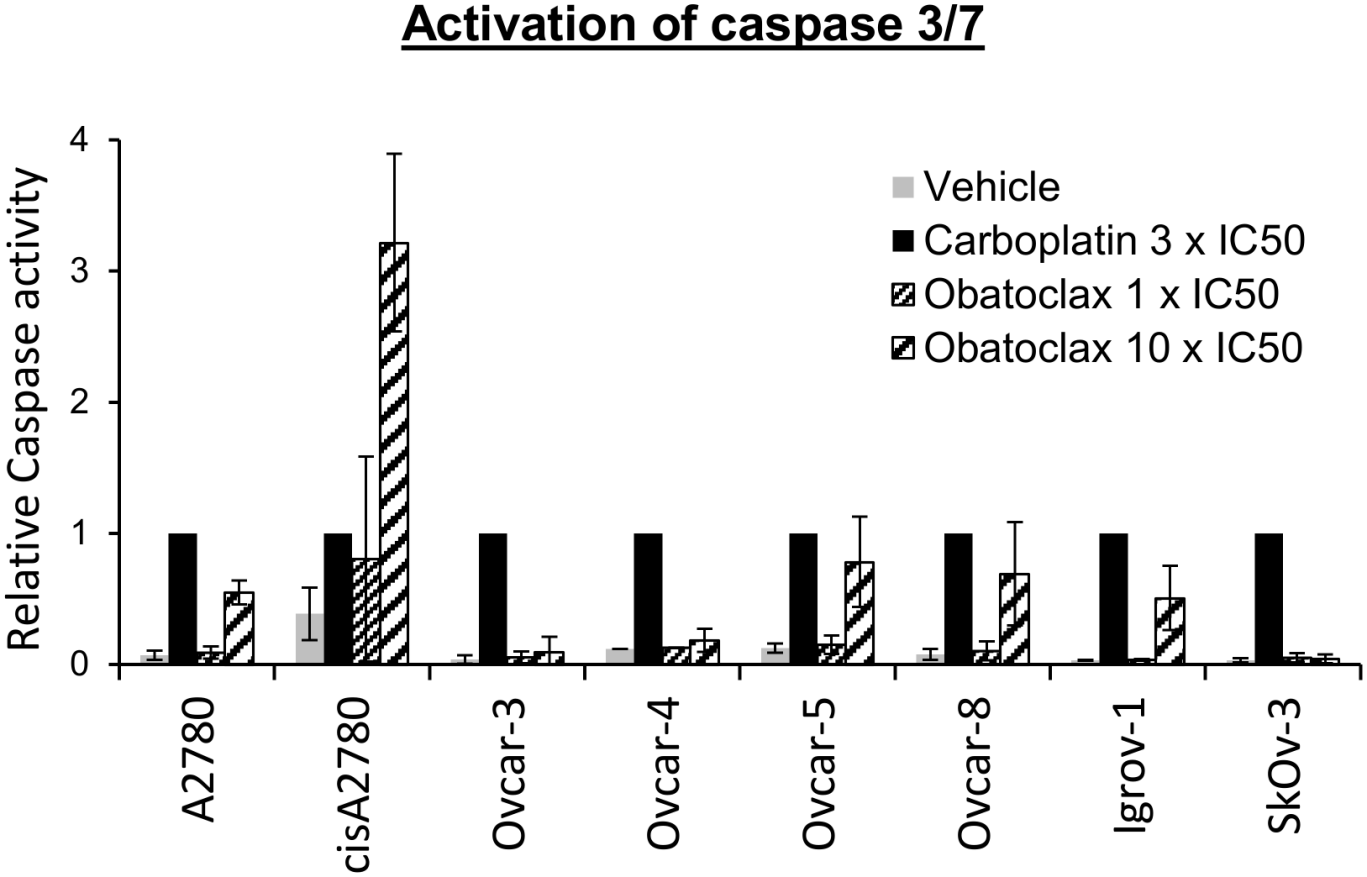
Obatoclax activates caspase 3/7. Eight ovarian cancer cell lines were treated with obatoclax for 30 hours.at the indicated multiple of the IC_50_ determined in cell proliferation assays (Table 1) Caspase 3/7 activity was measured (mean ± S.D., n = 3–5) and is expressed as a fraction of that observed in the same cell line exposed to carboplatin (as a positive control) for the same period and at a concentration equal to 3 x its IC_50_ in each cell line reported previously [3].

**Fig 4:**
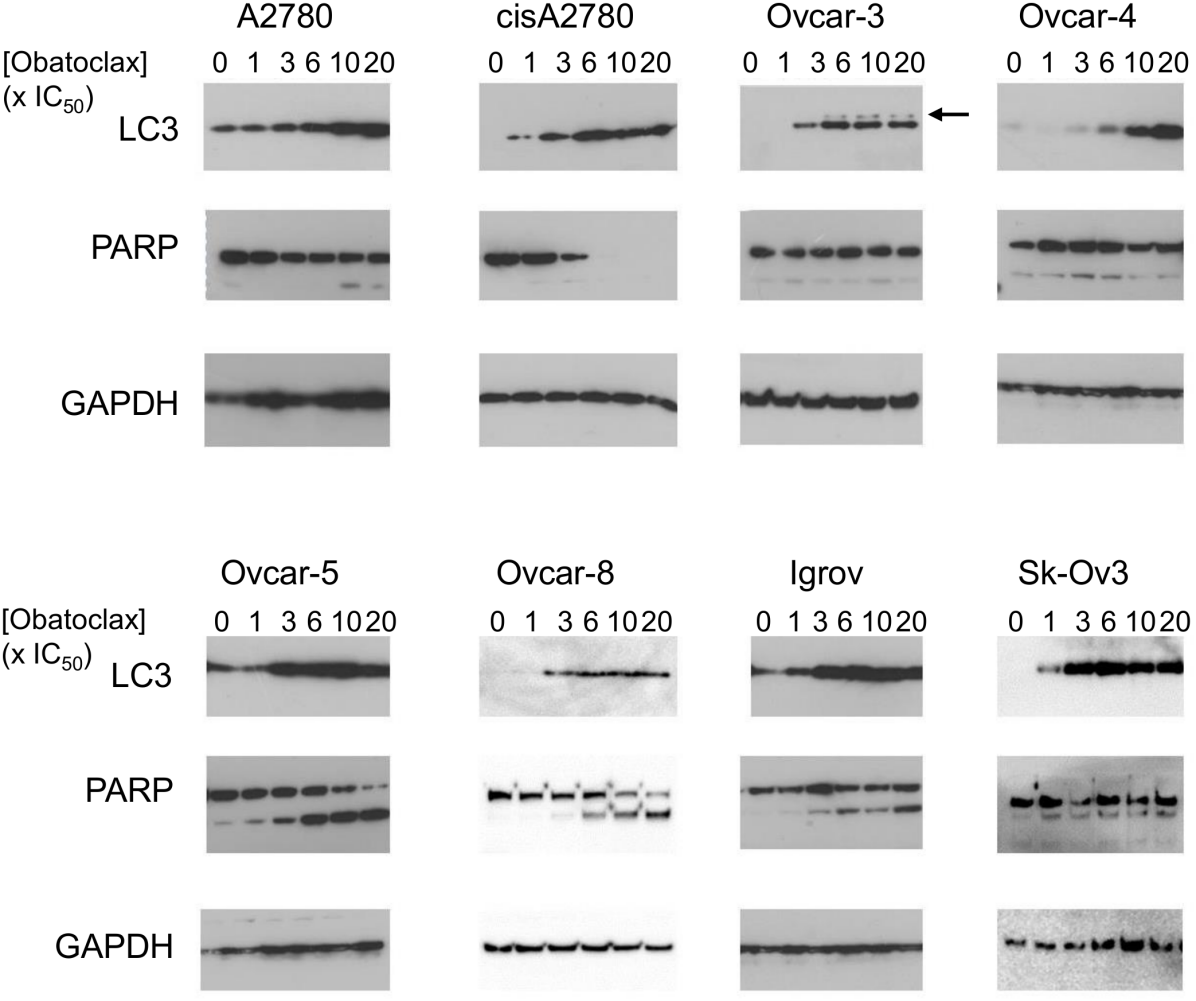
Obatoclax induces cleavage of PARP and accumulation of LC-3. Cells were treated with obatoclax for 48 hours at the indicated multiple of the IC_50_ determined in cell proliferation assays (Table 1). Lysates were prepared and PARP cleavage and accumulation of LC-3 was determined by immunoblotting. LC3-I and LC3-II were almost impossible to resolve in in most cell lines due to the very robust increase in LC3, although LC3-I (arrowed) and LC3-II could be distinguished in Ovcar-3 cells and obatoclax induced accumulation of LC3-II. The results shown are representative of 2–4 experiments per cell line.

Obatoclax inhibits all the Bcl-2 family members that regulate apoptosis. To test whether the cytotoxic activity of obatoclax reflects this, we determined the effect of obatoclax on the intrinsic apoptosis pathway. Increased caspase 3/7 activity (normalized to that induced by carboplatin) was observed in A2780, Ovcar-5, Ovcar-8 and Igrov-1 cells (Fig 3) and in these same cells cleavage of PARP was also apparent (Fig 4). (In cisA2780 cells there appeared to be a striking increase in caspase activity, normalized to that induced by carboplatin, but this reflects the relatively poor potency of carboplatin in this platinum-resistant cell line.) However, in Ovcar-3, Ovcar-4 and SkOv-3 cells carboplatin-normalized caspase-3/7 activity and PARP cleavage was relatively modest or undetectable and, notably, in these same cells, cytotoxicity measured by trypan blue staining in our previous experiments was also less pronounced (Fig 1). Taken together with the lack of synergy of obatoclax with carboplatin, these data suggested that obatoclax’s predominant mechanism of action was not as a BH3 mimetic and other mechanism(s) may contribute in some cell types. This prompted us to evaluate additional mechanisms of action. Obatoclax has been proposed to induce necroptosis or cell death through generation of reactive oxygen species. However, neither the necroptosis inhibitor necrostatin, N-acetylcysteine, nor the caspase inhibitor ZVAD FMK, substantially reduced cell death caused by obatoclax (S2 Fig).

**Fig 5:**
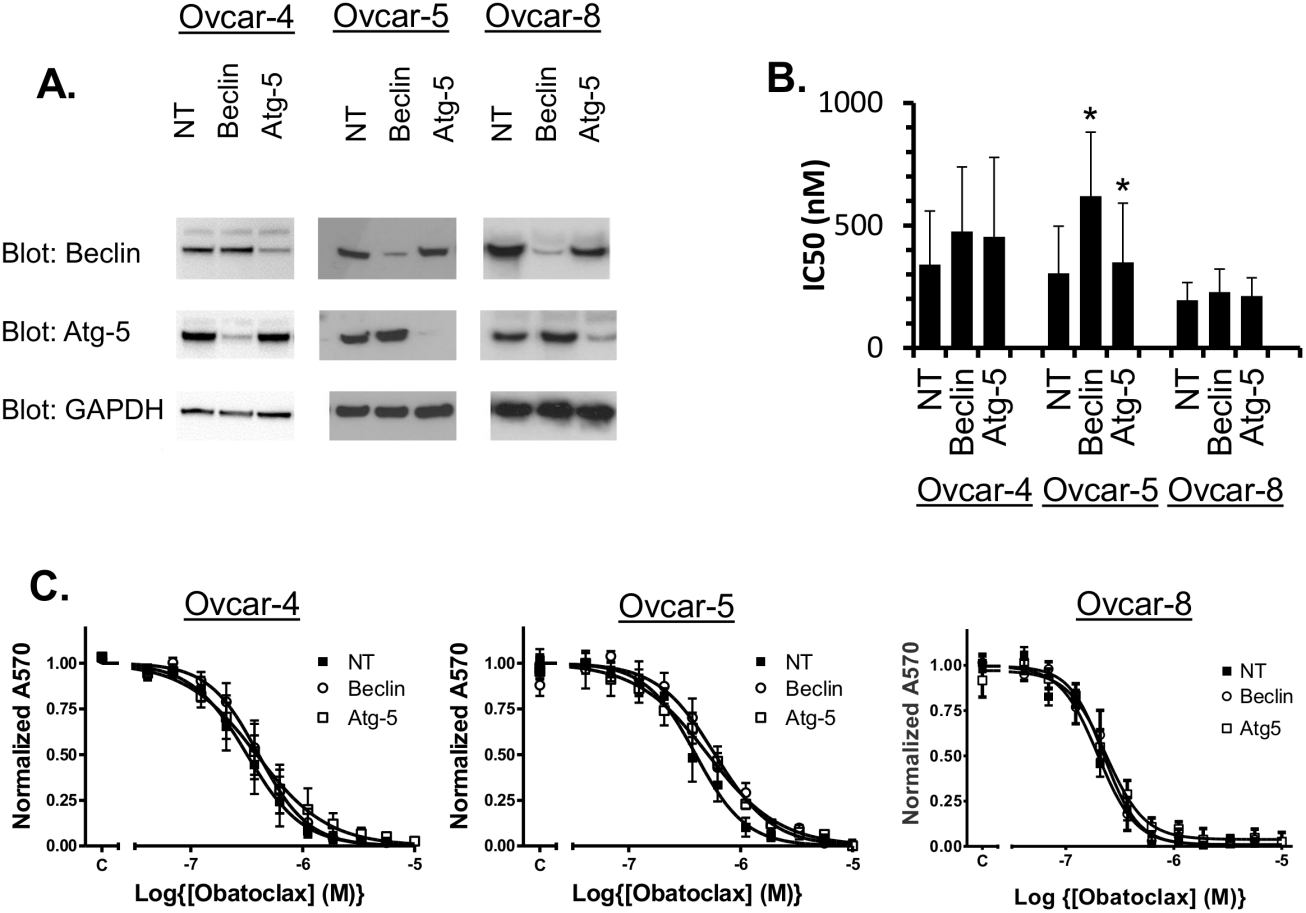
Effect of knockdown of Beclin-1 or Atg-5 on the activity of obatoclax. Ovcar-5 or Ovcar-8 cells were transfected with siRNA SMARTpools targeting Beclin-1 or Atg-5 or non-targeting siRNA (NT) and the effect on obatoclax activity determined in cell growth assays. **A**. Knockdown of Beclin-1 and Atg-5 was confirmed by western blotting 48 hours after transfection (representative of two experiments). **B**. 24 hours after transfection of siRNA SMARTpools targetting Atg-5 and Beclin-1, obatoclax was added and the IC_50_ of obatoclax (mean ± S.D., n = 3–4 separate transfections) determined and compared to that measured in cells transfected with a non-targeting (NT) siRNA. *, IC_50_ significantly different from that measured in cells transfected with NT siRNA (paired t- test, P < 0.05) C. Concentration-response data (mean ± S.D., n = 3–4) from which the IC_50_ values were determined.

Previous reports have claimed that obatoclax can induce autophagy based on the accumulation of LC-3. Consistent with this, obatoclax caused a robust increase in LC-3 immunoreactivity in all of the cell lines tested (Fig 4). In contrast, other work has suggested that obatoclax inhibits lysosomal function through an unknown mechanism [30] and consequently causing accumulation of LC-3. We used RNA interference to knock-down the expression of two key regulators genes of autophagy, Atg-5 and Beclin-1 and assessed the impact of this on the activity of obatoclax. In Ovcar-4, Ovcar-5 and Ovcar-8 cells, transfection with a pool of 4 siRNAs (Dharmacon SMARTpool) led to effective silencing of the expression of Atg-5 and Beclin-1 (Fig 5A). In Ovcar-5 cells a very modest reduction in potency of obatoclax was observed following knockdown of expression in Atg-5 and Beclin-1 (Fig 5B and 5C). However, the decreased expression of Atg5 and Beclin-1 did not significantly reduce the activity of obatoclax in Ovcar-4 or Ovcar-8 cells (Fig 5B and 5C). This suggests that activation of the autophagy pathway is not necessary for cell death induced by obatoclax.

**Fig 6:**
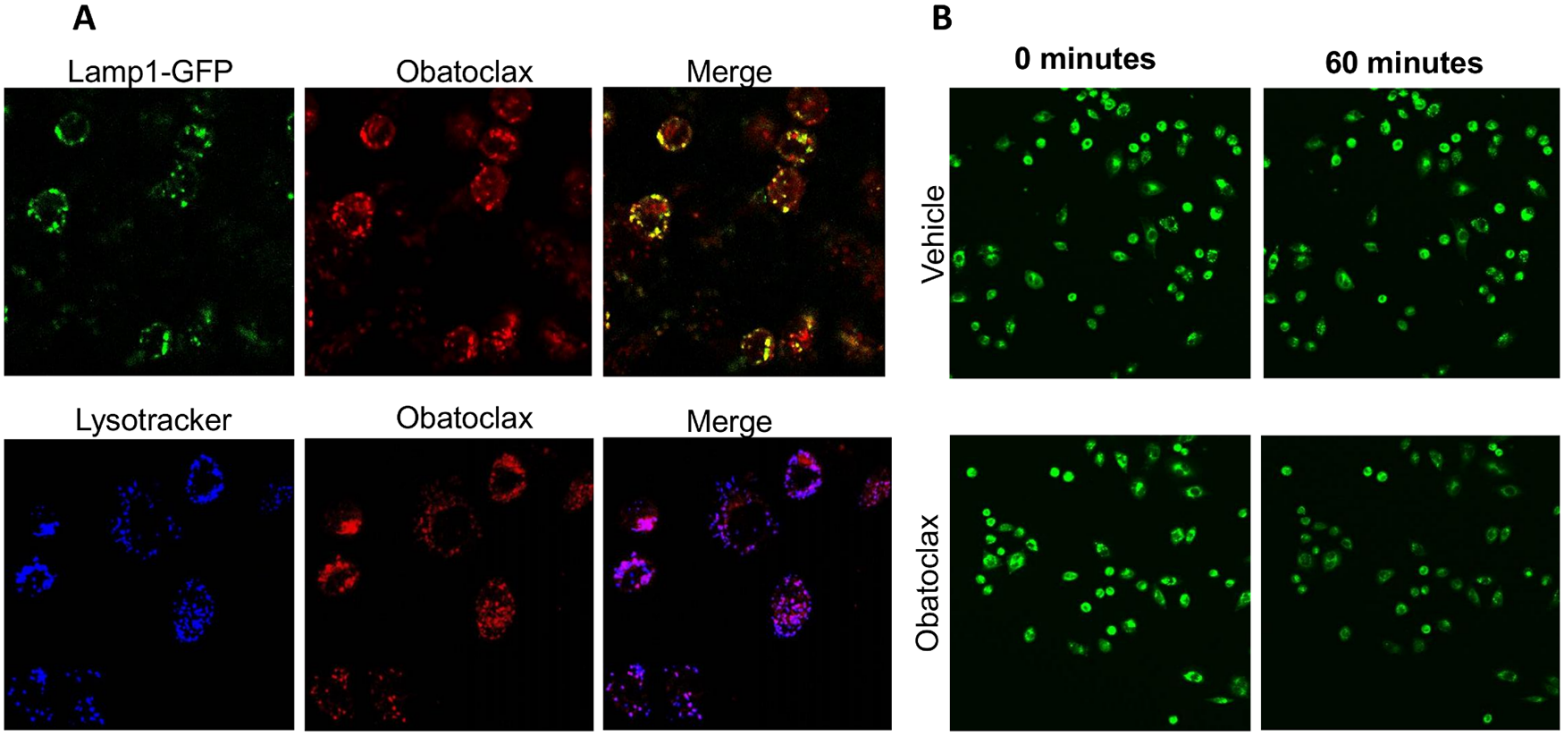
Obatoclax localizes to lysosomes and causes their alkalinization. **A**. Ovcar-5 cells expressing the acidic vesicle marker GFP-LAMP-1 (upper panel) or labelled with lysotracker blue dye (lower panel) were exposed to obatoclax for 1 hour. Obatoclax was measured by its intrinsic fluorescence. The cells were analysed by confocal microscopy and the results presented are representative of 3 experiments. **B**. Ovcar-5 Cells were labelled with lysosensor Green DND-189, exposed to 120 nM obatoclax for 1 hour. Images were captured at the indicated times. The decrease in fluorescence reflects alkalinization of the dye’s environment. The decrease in fluorescence is quantified in S5 Fig.

These observations raised the possibility that the activity of obatoclax was mediated by inhibition of lysosomal function although the mechanism by which obatoclax does this has not yet been reported. Basic drugs such as chloroquine can become protonated, charged and trapped in the acidic environments, causing their alkalinization. We noted that obatoclax contains two pyrrole moieties which would be expected to undergo protonation, become charged and possibly undergo similar ion-trapping. We made use of the intrinsic fluorescence of obatoclax [5] to monitor its subcellular localization. Cells were either treated with Bacmam particles encoding Lamp1 fused to GFP or with the lysotracker blue, both of which are markers for acidic organelles. Obatoclax was found to co-localize with both these markers (Fig 6A) demonstrating that obatoclax accumulates in acidic vesicles.

The presence of basic pyrrole groups in obatoclax suggested that its accumulation in lysosomes would render them more alkaline. To measure the effect of obatoclax on lysosomal pH we made use of lysosensor reagents whose fluorescence changes as a function of pH. To confirm that the lysosensor reagent can detect changes in lysosomal pH, Ovcar-5 cells were loaded with lysosensor green and exposed to the vATPase proton pump inhibitor bafilomycin, which is known to cause lysosomal alkalinization. As expected, bafilomycin caused a decrease in the lysosensor fluorescence (S3 Fig). To assess the effect of obatoclax on lysosomal pH, Ovcar-4, Ovcar-5 or Ovcar-8 cells were loaded with Lysosensor Green, exposed to obatoclax and changes in pH monitored by confocal microscopy. The decrease in fluorescence, which was rapid and evident within 15 minutes, suggested that obatoclax caused an increase in lysosomal pH (Fig 6B, S4 Fig and S1 Video). We quantitated the fluorescence change measured 15 minutes after the addition of obatoclax. Obatoclax caused a significant change in lysosensor green fluorescence in two cell lines which was dependent on the concentration of obatoclax used. We compared this to a calibration curve in which we determined the change in lysosensor blue fluorescence at different pHs *in vitro* and from this we estimated that obatoclax caused an increase in lysosomal pH of between 0.5 and 1 pH unit (S5 Fig). We considered the possibility that the decrease in lysosensor Green fluorescence might be caused by quenching by the accumulation of obatoclax in lysosomes. However, similar results were observed using a separate dye, Lysosensor yellow-blue which fluoresces at a significantly lower wavelength than lysosensor green (S6 Fig) and therefore less likely to undergo quenching by obatoclax. Additionally, the fluorescence (λ_EX_ = 456 nm, λ_Em_ = 528 nm) of a lysosensor green solution in sodium acetate (pH 5.3) in the presence of 5 μM obatoclax (40 times the concentrations used in the cell experiments) was 104 ± 6% (n = 4) of that measured in the absence of obatoclax, suggesting that obatoclax does not quench the fluorescence of the lysosensor reagent.

**Fig 7:**
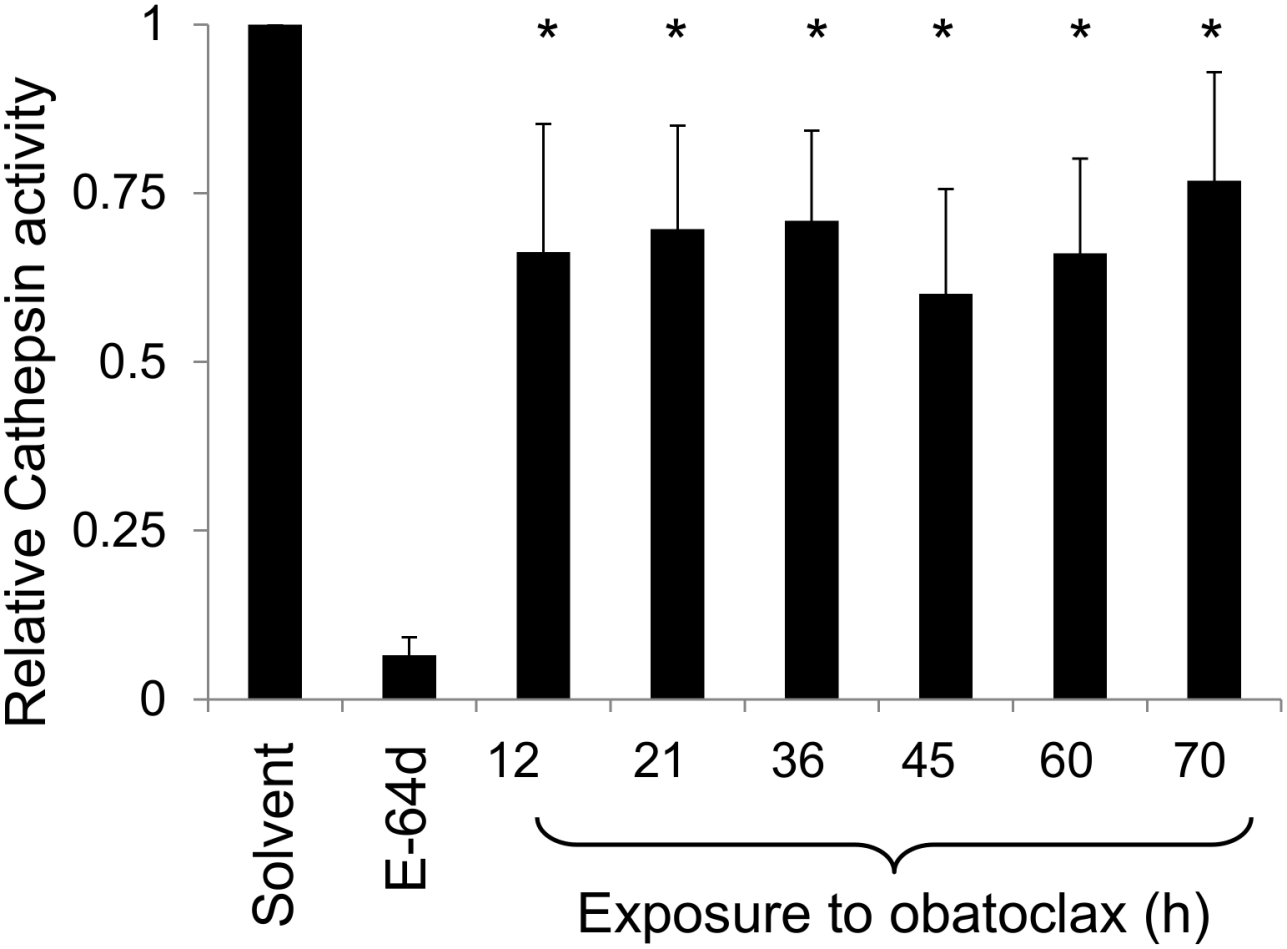
Obatoclax inhibits cathepsin activity in intact cells. Ovcar-5 cells were treated with solvent or obatoclax (120 nM, representing 3 x IC_50_ in the cell growth assay) for the indicated period. The cathepsin substrate Phe-Arg-AMC was added to the cells in serum-free medium for the last 12 hours of the incubation period. To confirm the assays measured cathepsin activity, cells were exposed to the cathepsin inhibitor E64d for the last 12 hours of the incubation period. After measuring fluorescence of the released AMC, the results were normalized to cell number by staining the cells with SRB. The results (means ± S.D., n = 4) are expressed as a fraction of the activity measured in cells treated with solvent alone and are significantly different from those measured in cells treated with solvent where indicated (“*”, t-test, P < 0.05).

To confirm that the increase in lysosomal pH altered lysosomal function, we measured cathepsin activity in cells treated with obatoclax. This experiment was performed with intact cells because measuring the activity in pH-buffered cell lysates would obscure the effect of lysosomal alkalinization on cathepsin activity. The hydrolysis of the cathepsin substrate Z-Phe-Arg-AMC was inhibited by 80% by the cathepsin inhibitor E64d. Within 12 hours of addition (the minimum assay duration necessary to detect accumulation of the fluorescent AMC product), obatoclax inhibited the hydrolysis of Z-Phe-Arg-AMC by approximately 30% (Fig 7) and this inhibition persisted up to 70 after drug addition.

**Fig 8:**
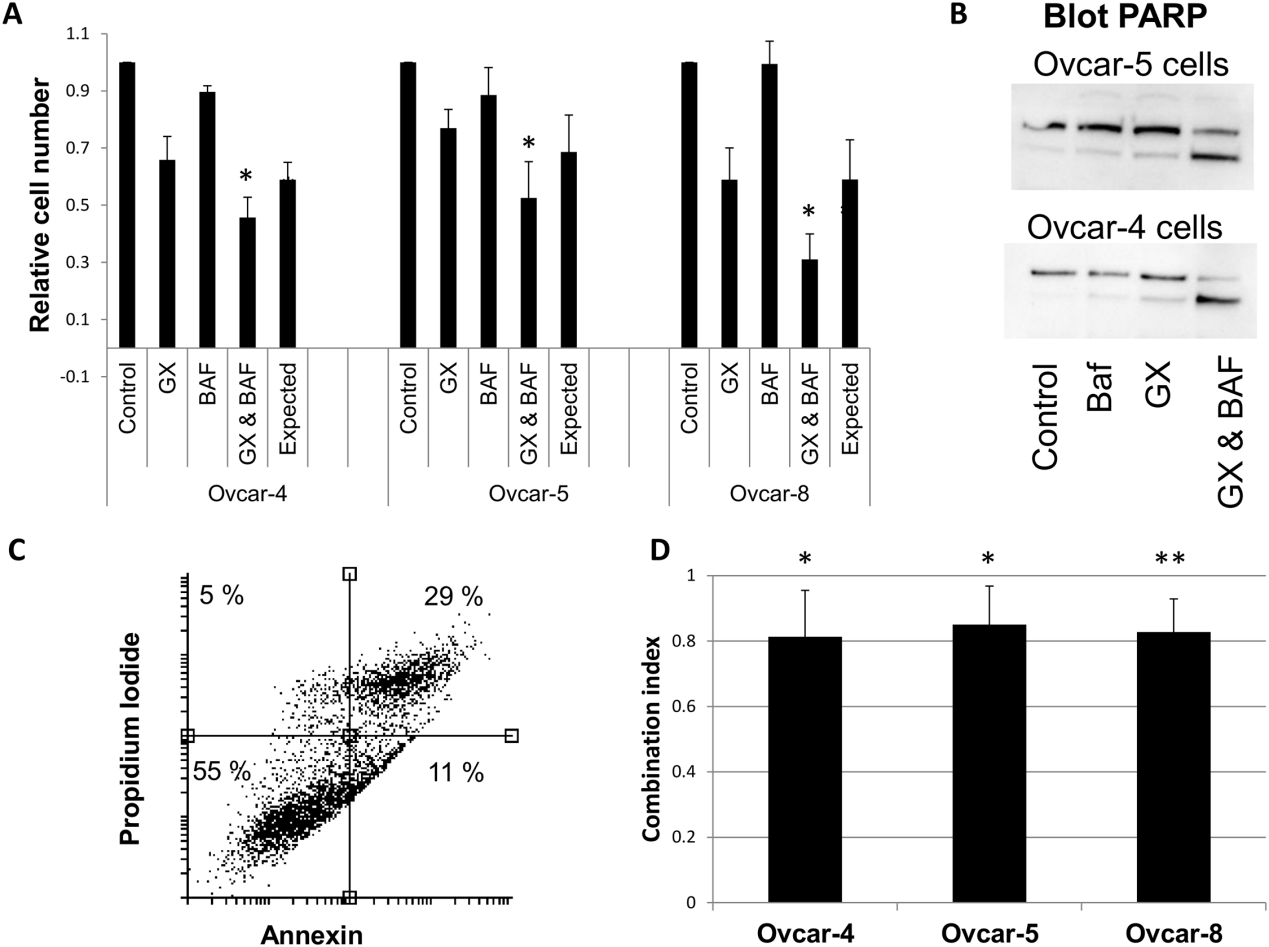
Obatoclax promotes cell death synergistically with other agents that cause lysosomal alkalinization. **A**. Ovcar-4, Ovcar-5 or Ovcar-8 cells were treated with obatoclax (“GX”, 3 x IC_50_), 10 nM bafilomycin (“BAF”) or a combination of both drugs for 72 hours. The number of cells surviving was determined by staining with SRB and was significantly different from the number expected from the Bliss independence criterion (“expected”) where indicated (“*”, t-test, P < 0.05). **B**. Ovcar-4 or Ovcar-5 cells were exposed to obatoclax (240 nM), bafilomycin (3nM) or a combination of both drugs for 48 h and PARP cleavage assessed by western blotting. (one experiment representative of 2–3). **C**. Ovcar-5 cells were exposed to obatoclax and bafilomycin as in C, stained with annexin V and propidium iodide and analysed by flow cytometery (the result shown is representative of 4 experiments). Only 6% of the cells treated with solvent stained positive for annexin V and propidium iodide. **D**. Ovcar-4, Ovcar-5 and Ovcar-8 cells were treated with obatoclax and chloroquine combined at a ratio of their respective IC_50_s using 18 different drug concentrations and the number of cells surviving after 3 days determined by staining with SRB. The combination index, calculated at fraction affected = 0.5, was significantly different from CI = 1.0 in all three cell lines (t- test, *, *P* < 0.05; **, *P* < 0.01)

These observations support the hypothesis that obatoclax accumulates in lysosomes and causes their alkalinization and raised the possibility that lysosomal alkalinization might contribute to the cytotoxic activity of obatoclax. It is difficult to design an experiment in which the effect of obatoclax on lysosomal pH is blocked to test this unambiguously. To overcome this, we hypothesized that if lysosomal alkalinization contributes to cell death induced by obatoclax, then obatoclax would synergize with other agents which cause cell death through lysosomal alkalinization. We evaluated the cytotoxic activity of obatoclax in combination with chloroquine, which causes lysosomal alkalinization by ion-trapping, or bafilomycin, which inhibits acidification of lysosomes by blocking the vacuolar ATPase that transports protons into the lysosome. When Ovcar-4, Ovcar-5 or Ovcar-8 cells were treated with bafilomycin and obatoclax in combination, a more than additive induction of cell death was observed (Fig 8A). Furthermore, the combination of these two agents led to an increase in PARP cleavage (Fig 8B) and an increasing the population of cells staining with both Annexin V and propidium iodide (Fig 8C), suggesting that this combination induced apoptosis. Similarly, when chloroquine and bafilomycin were combined at the ratio of their respective IC_50_s, a synergistic interaction was observed in cell growth assays (Fig 8D).

## Discussion

We have evaluated the mechanism by which obatoclax causes cell death. In contrast to our previous studies with the BH3 mimetics ABT-737 and navitoclax, obatoclax exhibited remarkable single agent potency in all 8 ovarian cancer cell lines tested and was not synergistic with chemotherapeutic agents. The latter observation suggests that functioning as a BH3 mimetic is not the predominant mechanism of action of obatoclax. This is significant because it suggests that, unlike authentic BH3 mimetics, combinations of obatoclax and/or paclitaxel are unlikely to be a successful strategy to treat ovarian cancer. In support of obatoclax having an additional non-BH3 mimetic mechanism of action, obatoclax caused cell death without marked apoptosis in a subset of the cell lines tested. In contrast, LC-3 accumulation was evident in all the cell lines tested but repression of Atg-5 and Beclin did not blunt the activity of obatoclax suggesting obatoclax blocked the end stages of autophagy. Obatoclax was found to accumulate in lysosomes and cause their alkalinization, explaining for the first time the recent report that obatoclax inhibits lysosomal function [30]. This activity is likely to contribute to the cell death induced by obatoclax because obatoclax was synergistic in cell growth/survival assays with two other agents, bafilomycin and chloroquine, which also cause lysosomal alkalinization.

Our data demonstrate that obatoclax, like chloroquine [36], accumulates in lysosomes. Obatoclax is an organic base, and so on entering the acidic environment of a lysosome it is likely to be protonated and positively charged. This charge may limit the diffusion of obatoclax from the lysosomes so it is effectively trapped in lysosomes—a process known as of ion-trapping. One of the earliest publications on obatoclax also noted that the drug accumulated in unidentifed intracellular “random puncta” [5]. Chloroquine’s activity as an antimalarial agent is dependent on its accumulation in the *Plasmodium* food vacuole as a result of ion-trapping, and it is notable that obatoclax also has anti-malarial activity [37], presumably also as a result of ion-trapping. Obatoclax is an organic base so its accumulation in lysosomes increases their pH. Lysosomes rely on an acidic environment to process material transported into them—for example, cathepsins are proteases with optimal activity at acidic pH and so obatoclax would be expected to interfere with lysosomal processing. Schwartz-Roberts and co-workers have shown that obatoclax reduces cathepsin levels and activity and cathepsin inhibitors can recapitulate some of the effects of obatoclax [30]. Both cathepsin inhibitors [30] and obatoclax [13, 29, 30] can also cause p62 and autophagosome accumulation. Previous workers [30] may have not detected a change in lysosomal pH following exposure to obatoclax because the longer drug exposure in their experiments led to an increase in total number of lysosomes and correspondingly increased staining with the lysosensor dye.

Obatoclax has also been described as a chloride ionophore [15] and it has been proposed that this contributes to its cytotoxic activity. Chloride is believed to be transported into lysosomes to compensate for the increased charge resulting from the accumulation of protons in lysosomes due to the activity of the vacuolar ATPase proton pump [38]. We cannot rule out the possibility that obatoclax also interferes with intracellular chloride gradients and that this also effects lysosomal pH, but this alone would not explain the accumulation of obatoclax in lysosomes.

**Fig 9:**
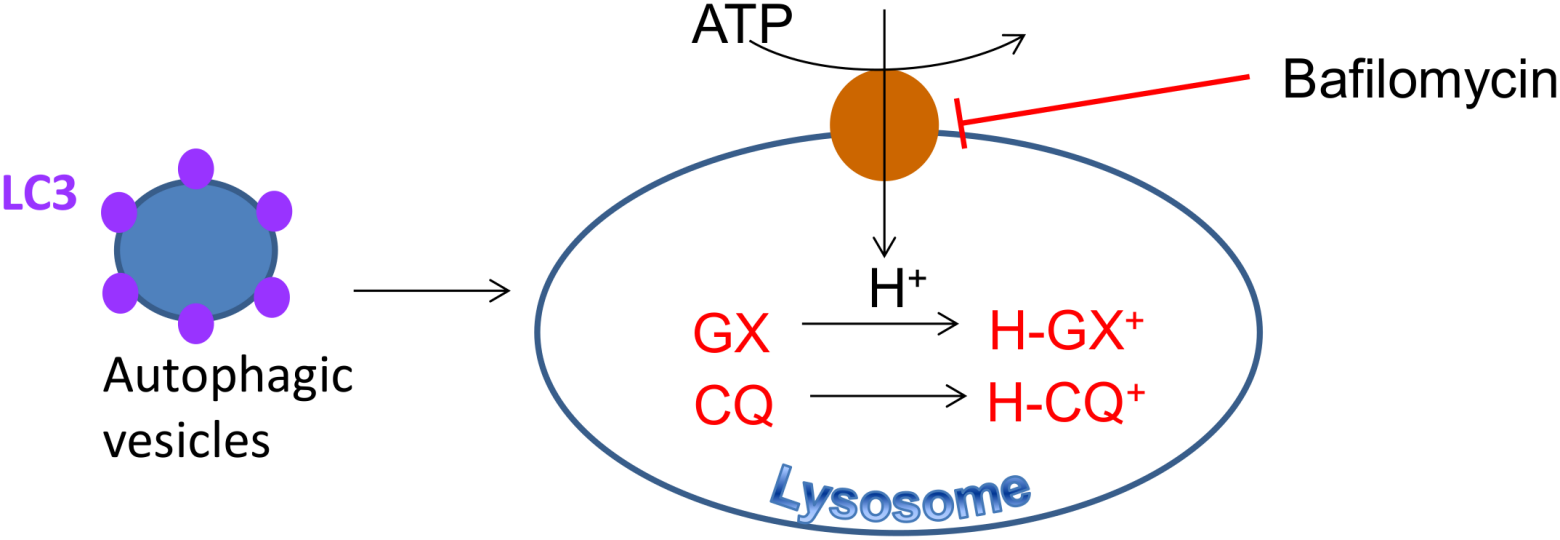
Model for the combined activity of obatoclax and chloroquine or bafilomycin. Bafilomycin blocks the vATPase reducing lysosomal H^+^ influx, while chloroquine (CQ) and obatoclax (OBX) are organic bases which become protonated and trapped in the lysosome and increase lysosomal pH. The resulting alkalinization reduces cathepsin activity as described [30] and the ensuing reduction in lysosomal function causes autophagosome and LC-3 accumulation.

The observation that obatoclax causes lysosomal alkalinization as well as cell death is a correlation and as such does not prove that this is a mechanism by which obatoclax exerts its cytotoxic effects. We found it difficult to design an experiment to address this unambiguously because the accumulation of obatoclax in lysosomes is likely to occur as a result of ion-trapping, a biophysical process. It is challenging to see how this process can be prevented, to demonstrate its role in obatoclax activity, without significantly compromising normal cell function. Furthermore, using obatoclax analogues that lack the pyrrole moieties that likely cause alkalinization is subject to the criticism that this could also interfere with other potential mechanisms of action. Instead, we hypothesized that if obatoclax was cytotoxic as a result of lysosomal alkalinization, it would be synergistic with other agents that increase lysosomal pH by reducing the intralysosomal proton concentration by other mechanisms. (Fig 9). We found that obatoclax was synergistic in cell growth/survival assays with bafilomycin and chloroquine and the combination induced apoptosis. Synergy between chloroquine and obatoclax has also been observed in pancreatic cancer cells [19]. It is also noteworthy that both bafilomycin and archazolid, inhibitors of the vacuolar ATPase, both cause lysosomal alkalinization and cell death [39, 40]. Taken together, these data are strongly supportive of the hypothesis that lysosomal alkalinization contributes to cell death induced by obatoclax but they do not demonstrate it unequivocally. The change in lysosomal pH occurred rapidly (within 15 minutes) and the inhibition of cathepsin activity was observed at the earliest time point we could measure, whereas substantial cell death was not evident until 48 hours. This suggests that impaired lysosomal function may cause cumulative defects in metabolism (eg through inhibition of autophagy) which contributes to cell death.

This proposed mechanism of action of obatoclax can explain much of the conflicting literature regarding this molecule. Several reports have suggested that obatoclax can induce autophagy based on the increase in LC-3 and the accumulation of autolysosomes/ autophagosomes [13, 29, 30]. In contrast, our data suggest that these observations can, at least in part, be explained by the inhibition of the end stage of autophagy as a result of lysosomal alkalinization leading to the well documented [13, 29, 30] accumulation of LC-3 and autophagosomes. Obatoclax, in combination with flavopiridol, has been reported to induce apoptosis through the accumulation of BIK [41] and it is possible that inhibition of lysosomal function contributes to this by reducing BIK turnover. Obatoclax also has been reported to induce necroptosis through the assembly of a necrosome on autophagic vesicles [42]. This may result from the blockade of lysosomal function and increase in autophagosome number caused by obatoclax promoting the assembly of necrosomes. However, in in our experiments necrostatin failed to block the cytotoxic activity of obatoclax. Combinations of kinase inhibitors and obatoclax have also been reported to induce “toxic autophagy” [27, 43]. We suggest that this is, at least in part, due to inhibition of lysosomal function. However, obatoclax has been shown to activate AMPK [14] and disrupt the complex between beclin1 and Mcl-1 [25], events linked to activation of the autophagy pathway. Furthermore studies with GFP and RFP dual-tagged LC3, as well as studies measuring LC-3 in cells treated with obatoclax and/or bafilomycin, have suggested that obatoclax stimulates autophagy [29]. Thus, we cannot rule out the possibility that in some cells obatoclax both stimulates the early stages and inhibits the end-stage of autophagy and that these opposing effects lead to the profound increase in autophagosomes and LC-3. However, our data argue against a substantial contribution from stimulating autophagic flux to cell death in the cell lines we have evaluated because inhibition of the expression of Atg-5 or Beclin-1 does not have a substantial effect on the activity of obatoclax. Notably, repression of Atg-7 also does not substantially affect the activity of obatoclax even in cells lacking and Bak and Bax [10].

Considering both previously published studies of obatoclax and our data together, we suggest that studies with obatoclax do not provide evidence for a death-inducing form of autophagy, as has been proposed [31], rather they demonstrate that cell death can ensue from inhibition of the end-stage of the autophagy pathway as a result of lysosomal alkalinization. This is likely to be supplemented by the effects of obatoclax on the intrinsic apoptosis pathway. Indeed, in our studies, obatoclax clearly induced apoptosis in several cell lines. These processes may be linked, because the inhibition of autophagy can lead to the accumulation of pro-apoptotic BH3-only protein BIK as a result of failure to load cargo into autophagosomes [41]. Indeed, when we combined obatoclax with bafilomycin, PARP cleavage was observed in Ovcar-4 cells which normally did not undergo apoptosis in response to obatoclax alone.

Why some cells undergo apoptosis and others a non-apoptotic form of cell death after exposure to obatoclax is unclear. One possibility is that obatoclax is able to cause greater alkalinization in some cells, perhaps due to differences in expression of vATPase. Differences in basal autophagic flux through the pathway are also likely to affect the extent of accumulation of autophagosomes after obatoclax blocks their degradation and this may explain the variable effects of the knockdown of *ATG* genes on the activity of obatoclax reported in the literature. Alternatively, in cells in which there are pre-existing complexes of Bcl-2 family inhibitors primed with pro-apoptotic BH3-only proteins [44], apoptosis may be favored. We hypothesize that that in cells in which pro-apoptotic BH3-only proteins are constitutively produced, obatoclax can either displace them from Bcl-2 proteins (by acting as a BH3 mimetic) or by blocking their autophagic turnover (through lysosomal alkalinization), and consequently induce apoptosis. We also hypothesize that in cells lacking constitutive BH3-only protein production, cell death ensues as a result of blockade of lysosomal function. Further studies will be necessary to demonstrate this.

We conclude that obatoclax accumulates in lysosomes, causes their alkalinization and that this explains the inhibition of lysosomal function reported previously [30]. It is likely that in many cell types this is a major mechanism by which it exerts it cytotoxic effects, although in some cells apoptosis is still observed.

## Supporting Information

S1 Fig Effect of obatoclax on the cell cycle. Cells were treated with obatoclax for 48 hours at the multiple of the IC_50_ determined in cell proliferation assays (Table 1). Cells were harvested, fixed and stained with propidium iodide prior to analysis by flow cytometry. The results are the average of 3–4 experiments with each cell line.
(TIF)

S2 Fig Effect of ZVAD, necrostatin and N acetylcysteine on the activity of obatoclax in cell growth assays. Ovcar 4, Ovcar-5 or Ovcar-8 cells were treated with obatoclax (3 x IC50 reported in Table 1) and either vehicle or ZVAD (20 μM), necrostatin (5 μM) or N-acetylcysteine (10 mM) and after 72 hours the surviving cell number determined by staining with SRB. The result are presented as the fraction of the SRB stain measured in samples treated with vehicle alone (mean ± S.D., n = 4).
(TIF)

S3 Fig Bafilomycin decreases fluorescence of lysosensor green in Ovcar-5 cells. Ovcar-5 cells were loaded with lysosensor green, and treated with vehicle or bafilomycin (100nM) for 1 hour before images were captured by confocal microscopy. The results are representative of two experiments. The decrease in fluorescence reflects alkalinization of the dye’s environment.
(TIF)

S4 Fig Alkalinization of lysosomes in Ovcar-8 cells by obatoclax measured by lysosensor green. Ovcar-8 cells were labelled with lysosensor Green DND-189, and exposed to vehicle or 120 nM obatoclax for 1 hour. The decrease in fluorescence reflects alkalinization of the dye’s environment.
(TIF)

S5 Fig Quantification of the change in fluorescence of lysosensor green in cells treated with obatoclax. **A**. The fluorescence (λ_Ex_ = 420 nm, λ_Em_ = 550 nm) of lysosensor green was measured in vitro using a series of pH 4.2 to pH 6.5 sodium acetate (100 mM) buffered solutions. The results were normalized to maximum fluorescence observed at pH 5. **B**. Ovcar-5 or Ovcar-8 cells loaded with lysosensor green and images captured before and 15 minutes after the addition of the indicated concentration of obatoclax. The changes in fluorescence, expressed as a percentage of that observed prior to drug addition, were quantified to determine average pixel intensity using Olympus Fluoview software (*, significantly different to value prior to drug addition, t-test, *P* < 0.05). Comparision to the calibration curve (A) and assuming an initial lysosomal pH of 4.5–5.0, suggests that the approximately 25% reduction in fluorescence corresponds to increase of pH between 0.5 and 1 pH unit.
(TIF)

S6 Fig Alkalinization of lysosomes in Ovcar-5 cells by obatoclax measured with lysosensor yellow-blue. Ovcar-5 cells were labelled with lysosensor yellow blue DND-160 and exposed to 120 nM. Although this dye allows ratiometric imaging, we found that the longer wavelength portion of the emission spectrum overlapped significantly with that of obatoclax. Consequently, the images show the decrease in blue fluorescence accompanying decreased lysosomal pH. We separately confirmed that the fluorescence (λ_Ex_ = 405, λ_Em_ = 440) of a lysosensor yellow blue DND-160 solution decreased at alkaline pH and at pH 9 was 19 ± 1% (n = 4, mean ± S.D.) of that measured at pH 3.0.
(TIF)

S1 Video Ovcar-5 cells were loaded with lysosensor green and exposed to vehicle or 120 nM obatoclax. Still images were captured every minute for 1 hour and these images converted into a video. The video represents 70 min of real time, compressed 750 fold.
(WMV)
