# Supplementary figures and images for: The BH3 Mimetic Obatoclax Accumulates in Lysosomes and Causes Their Alkalinization
Source: PLoS One. 2016 Mar 7;11(3):e0150696. doi: 10.1371/journal.pone.0150696 (PMC4780728; doi:10.1371/journal.pone.0150696)

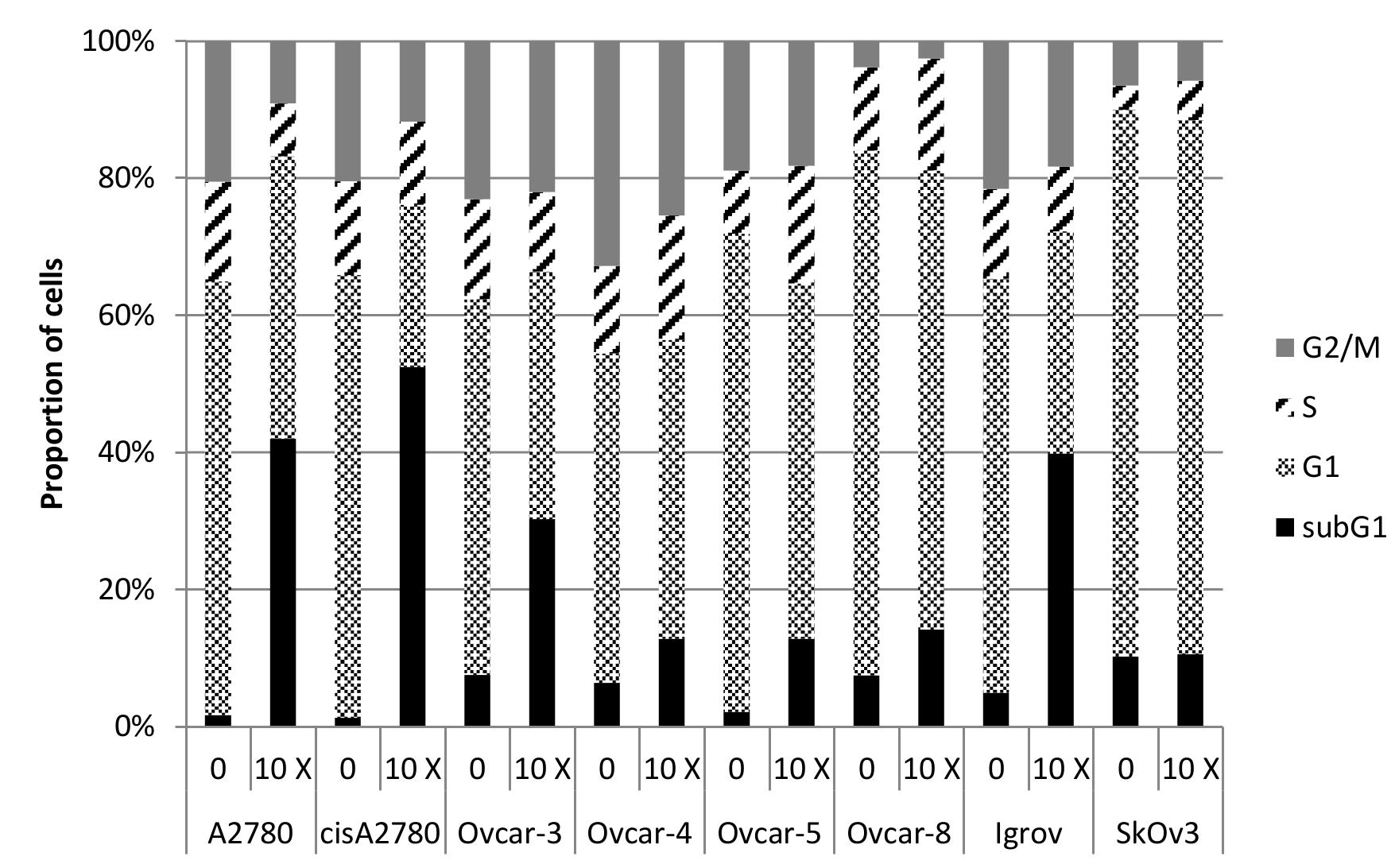

Supplement: S1 Fig — Cells were treated with obatoclax for 48 hours at the multiple of the IC50 determined in cell proliferation assays (Table 1). Cells were harvested, fixed and stained with propidium iodide prior to analysis by flow cytometry. The results are the average of 3–4 experiments with each cell line. (TIF) [file pone.0150696.s001.tif]

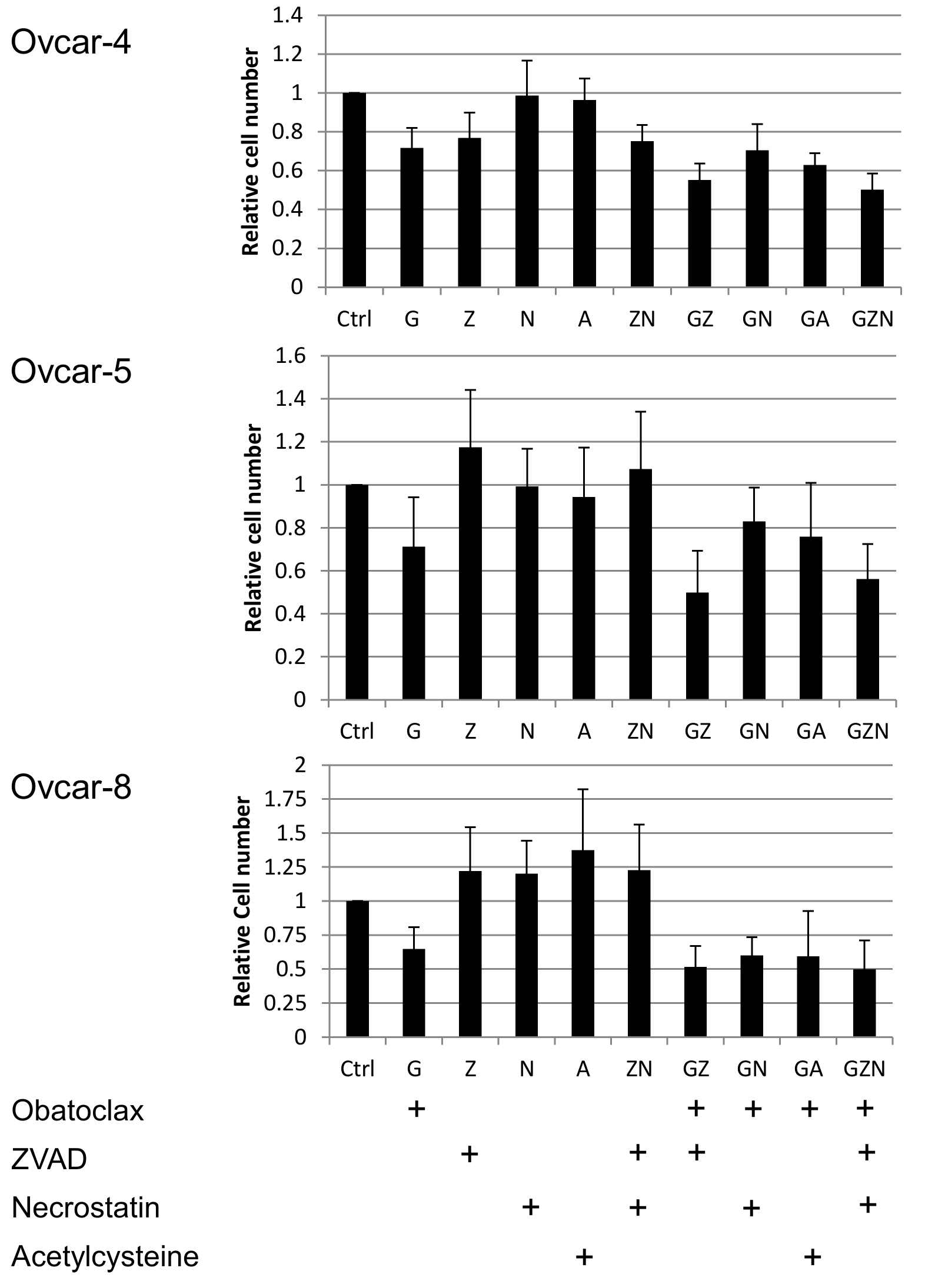

Supplement: S2 Fig — Ovcar 4, Ovcar-5 or Ovcar-8 cells were treated with obatoclax (3 x IC50 reported in Table 1) and either vehicle or ZVAD (20 μM), necrostatin (5 μM) or N-acetylcysteine (10 mM) and after 72 hours the surviving cell number determined by staining with SRB. The result are presented as the fraction of the SRB stain measured in samples treated with vehicle alone (mean ± S.D., n = 4). (TIF) [file pone.0150696.s002.tif]

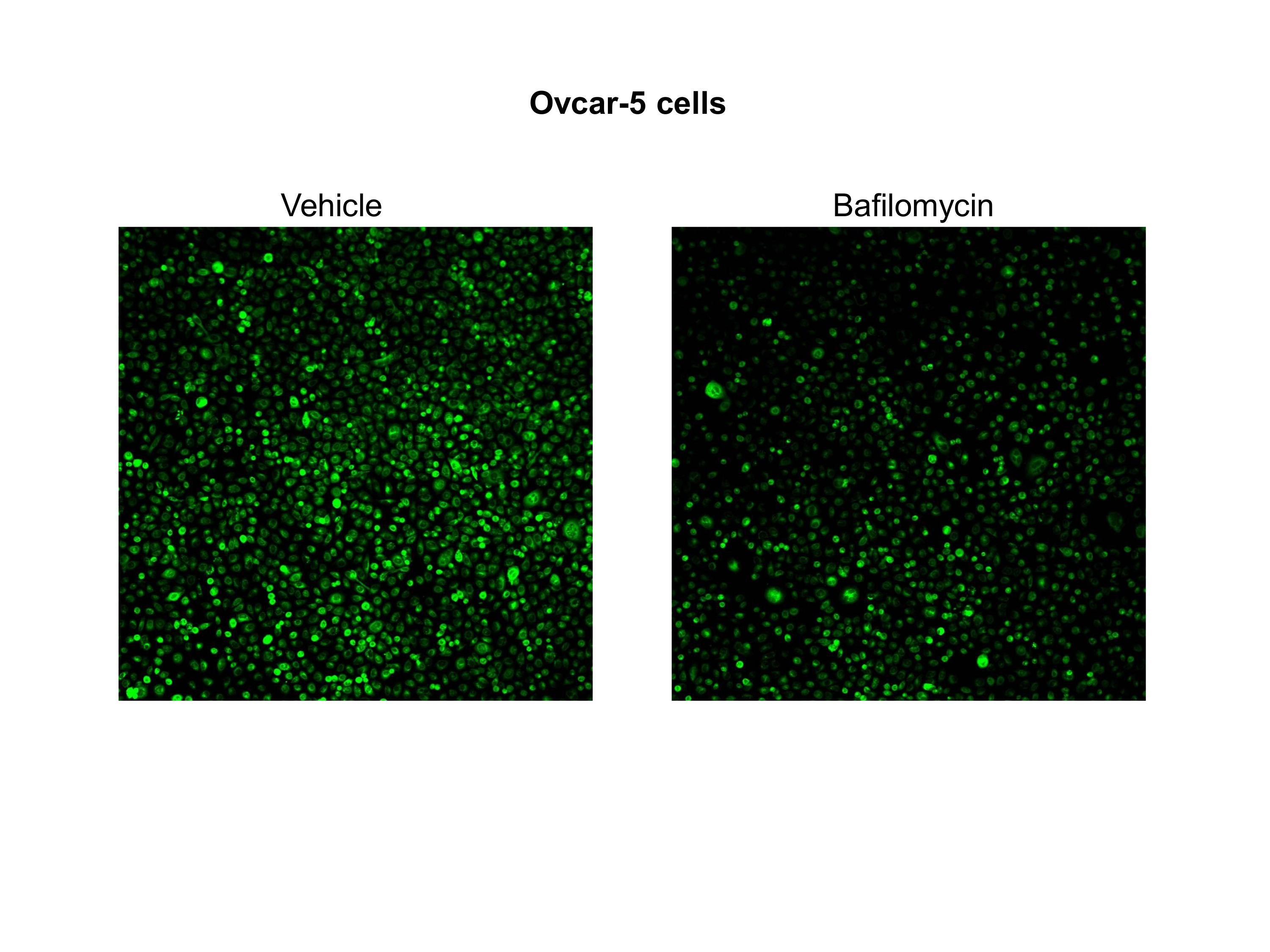

Supplement: S3 Fig — Ovcar-5 cells were loaded with lysosensor green, and treated with vehicle or bafilomycin (100nM) for 1 hour before images were captured by confocal microscopy. The results are representative of two experiments. The decrease in fluorescence reflects alkalinization of the dye’s environment. (TIF) [file pone.0150696.s003.tif]

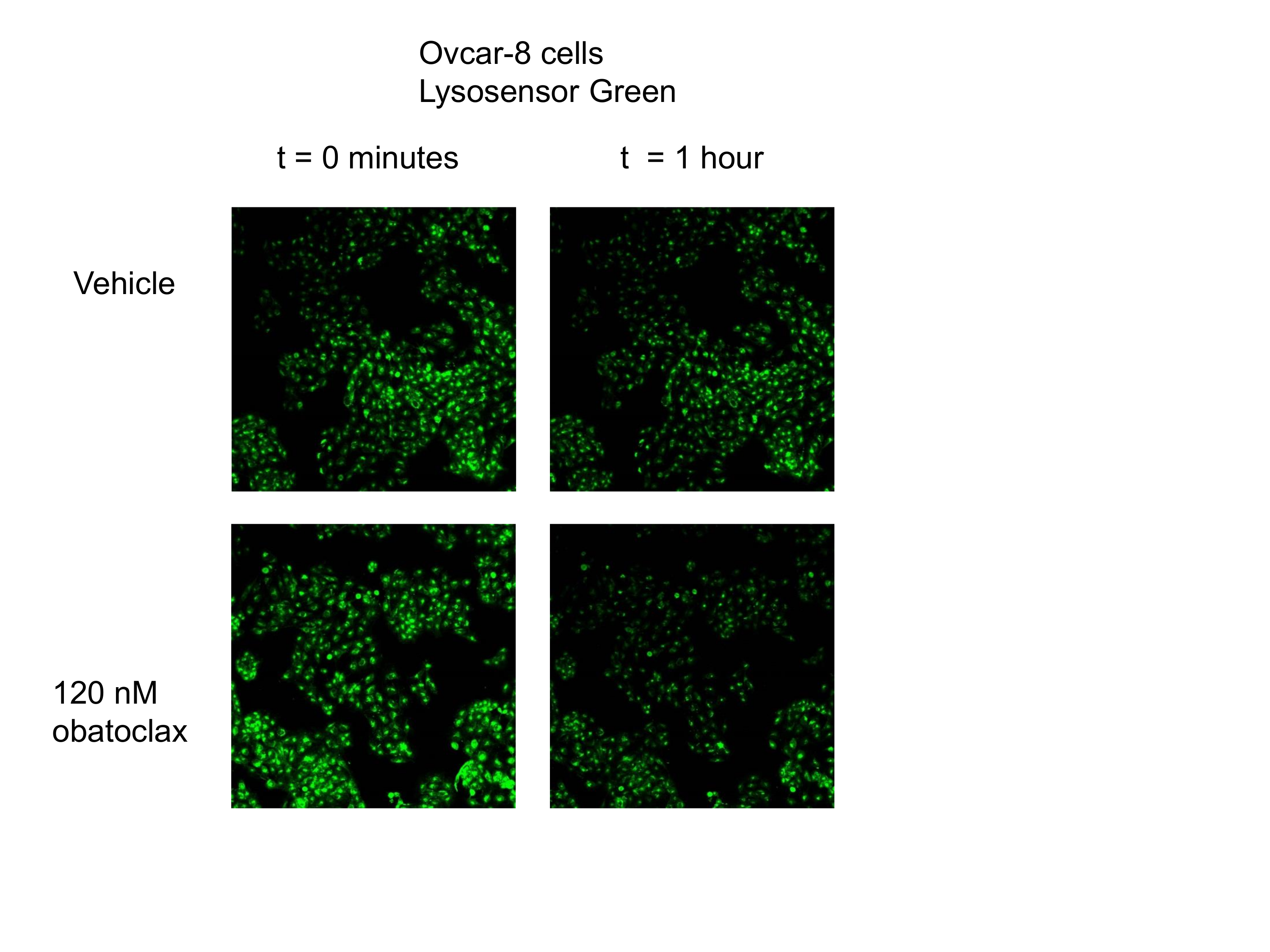

Supplement: S4 Fig — Ovcar-8 cells were labelled with lysosensor Green DND-189, and exposed to vehicle or 120 nM obatoclax for 1 hour. The decrease in fluorescence reflects alkalinization of the dye’s environment. (TIF) [file pone.0150696.s004.tif]

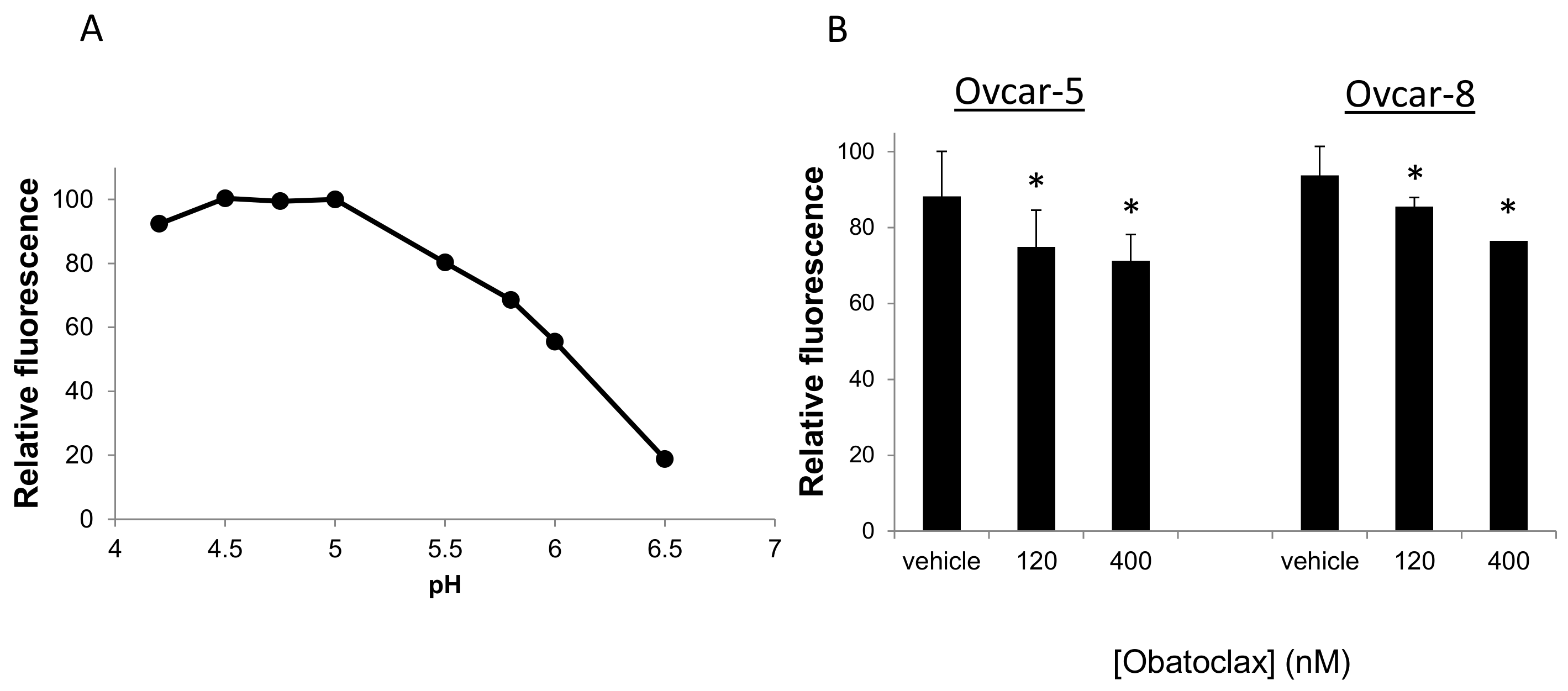

Supplement: S5 Fig — A. The fluorescence (λEx = 420 nm, λEm = 550 nm) of lysosensor green was measured in vitro using a series of pH 4.2 to pH 6.5 sodium acetate (100 mM) buffered solutions. The results were normalized to maximum fluorescence observed at pH 5. B. Ovcar-5 or Ovcar-8 cells loaded with lysosensor green and images captured before and 15 minutes after the addition of the indicated concentration of obatoclax. The changes in fluorescence, expressed as a percentage of that observed prior to drug addition, were quantified to determine average pixel intensity using Olympus Fluoview software (*, significantly different to value prior to drug addition, t-test, P < 0.05). Comparision to the calibration curve (A) and assuming an initial lysosomal pH of 4.5–5.0, suggests that the approximately 25% reduction in fluorescence corresponds to increase of pH between 0.5 and 1 pH unit. (TIF) [file pone.0150696.s005.tif]

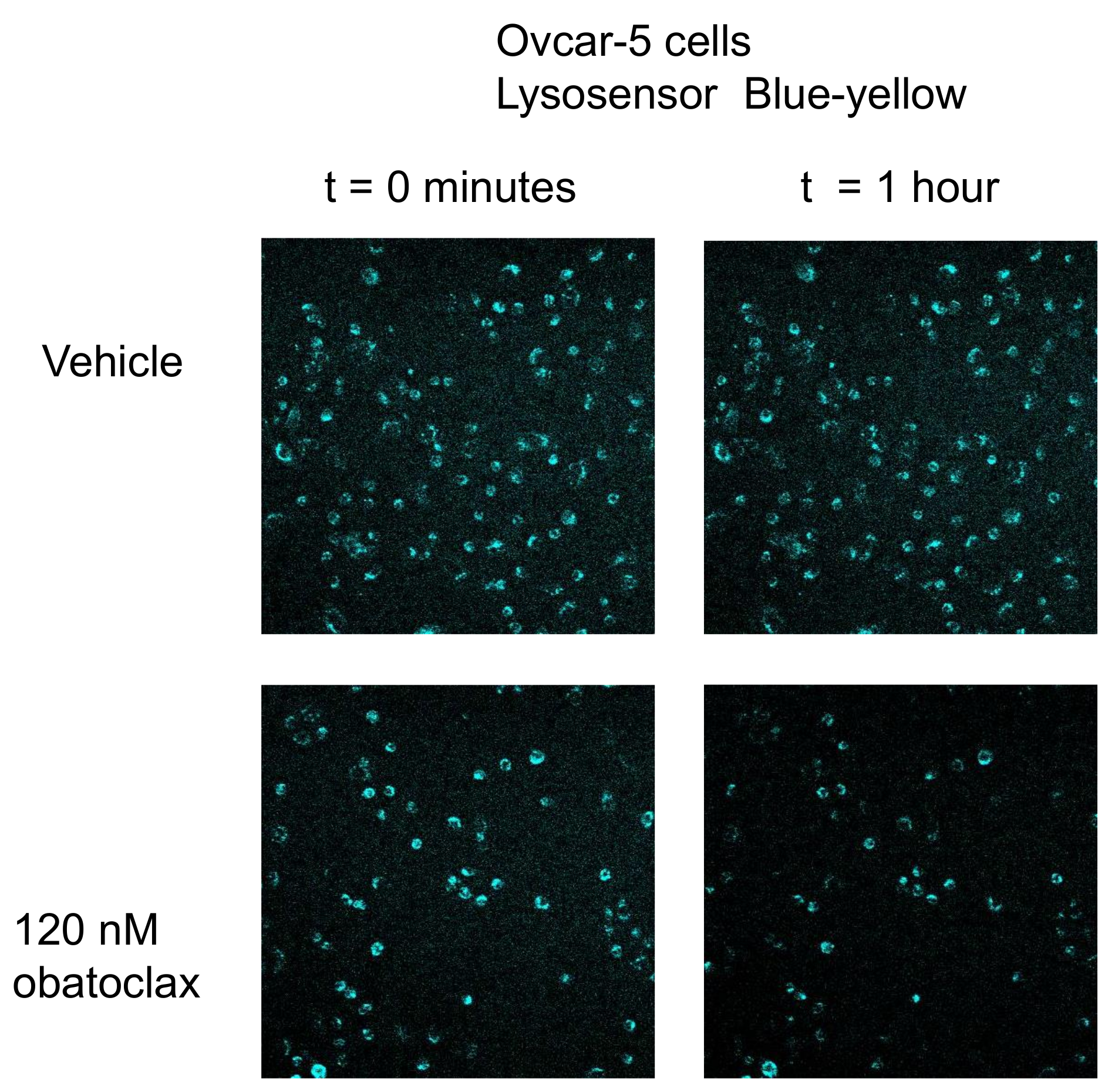

Supplement: S6 Fig — Ovcar-5 cells were labelled with lysosensor yellow blue DND-160 and exposed to 120 nM. Although this dye allows ratiometric imaging, we found that the longer wavelength portion of the emission spectrum overlapped significantly with that of obatoclax. Consequently, the images show the decrease in blue fluorescence accompanying decreased lysosomal pH. We separately confirmed that the fluorescence (λEx = 405, λEm = 440) of a lysosensor yellow blue DND-160 solution decreased at alkaline pH and at pH 9 was 19 ± 1% (n = 4, mean ± S.D.) of that measured at pH 3.0. (TIF) [file pone.0150696.s006.tif]
